# Perspective on the integration of optical sensing into orthopedic surgical devices

**DOI:** 10.1117/1.JBO.27.1.010601

**Published:** 2022-01-05

**Authors:** Carl Fisher, James Harty, Albert Yee, Celina L. Li, Katarzyna Komolibus, Konstantin Grygoryev, Huihui Lu, Ray Burke, Brian C. Wilson, Stefan Andersson-Engels

**Affiliations:** a Biophotonics@Tyndall, IPIC, Tyndall National Institute, Lee Maltings, Dyke Parade, Cork, Ireland; b Cork University Hospital and South Infirmary Victoria University Hospital, Department of Orthopaedic Surgery, Cork, Ireland; c University of Toronto, Sunnybrook Research Institute, Department of Surgery, Holland Bone and Joint Program, Division of Orthopaedic Surgery, Sunnybrook Health Sciences; Orthopaedic Biomechanics Laboratory, Physical Sciences Platform, Toronto, Canada; d University of Toronto, Princess Margaret Cancer Centre/University Health Network, Department of Medical Biophysics, Toronto, Canada; e University College Cork, Department of Physics, Cork, Ireland

**Keywords:** optics, surgery, orthopedics, guidance, biophotonics

## Abstract

**Significance:**

Orthopedic surgery currently comprises over 1.5 million cases annually in the United States alone and is growing rapidly with aging populations. Emerging optical sensing techniques promise fewer side effects with new, more effective approaches aimed at improving patient outcomes following orthopedic surgery.

**Aim:**

The aim of this perspective paper is to outline potential applications where fiberoptic-based approaches can complement ongoing development of minimally invasive surgical procedures for use in orthopedic applications.

**Approach:**

Several procedures involving orthopedic and spinal surgery, along with the clinical challenge associated with each, are considered. The current and potential applications of optical sensing within these procedures are discussed and future opportunities, challenges, and competing technologies are presented for each surgical application.

**Results:**

Strong research efforts involving sensor miniaturization and integration of optics into existing surgical devices, including K-wires and cranial perforators, provided the impetus for this perspective analysis. These advances have made it possible to envision a next-generation set of devices that can be rigorously evaluated in controlled clinical trials to become routine tools for orthopedic surgery.

**Conclusions:**

Integration of optical devices into surgical drills and burrs to discern bone/tissue interfaces could be used to reduce complication rates across a spectrum of orthopedic surgery procedures or to aid less-experienced surgeons in complex techniques, such as laminoplasty or osteotomy. These developments present both opportunities and challenges for the biomedical optics community.

## 1 Introduction

In most developed countries, orthopedic surgery represents one of the most common surgical procedures.^1^ The number of patients and procedures will only increase with aging populations. Many surgical procedures have been developed successfully to treat common degenerative conditions, including total knee or hip replacement and spinal fusion, which are the most common hospital discharge procedures in the United States with about 1.5 million cases per year. Other procedures, such as partial and total shoulder replacement, partial hip replacement, kyphoplasty/vertebroplasty, and lumbar decompression, are also regularly performed by orthopedic surgeons as well as other specialists (neurosurgeons and interventional radiologists). Minimally invasive surgery (MIS) and percutaneous techniques have gradually been introduced to reduce complications and improve recovery times:^2^^–^^4^ examples in spinal surgery include pedicle screw placement, spinal fusion, vertebroplasty/kyphoplasty, and decompression. There has been more recent adoption in total hip arthroplasty, with renewed interest in anterior hip approaches.^5^^–^^7^ Other hip arthroplasty techniques have included bone resurfacing, which has been controversial with several large registries highlighting concerns over premature wear and metallosis.^8^^,^^9^

For minimally invasive approaches, adjunctive imaging is used frequently to plan and guide procedures, including endoscopy,^10^^,^^11^ x-ray fluoroscopy or CT scanning,^12^^–^^15^ infrared navigation with visualization^3^^,^^4^^,^^16^^–^^18^ and robotics with endoscopy.^19^^,^^20^ The overall goal is to provide information on the spatial position of surgical tools in either a two-dimensional plane or three-dimensional volume or direct visualization of the surgical area during the procedure to minimize surgical complications such as pedicle breach in percutaneous pedicle screw fixation. Intraoperative ultrasound is also being used increasingly via keyhole spinal surgery to guide indirect decompression of disc, bone and other pathologies that cause neurologic compression. In open surgery, imaging techniques such as fluoroscopy may be used in total hip revision to facilitate femoral cement removal, especially at the distal end where low visibility is accompanied by risk of shaft fracture and mutilation.^21^^,^^22^ Infrared navigation and instrument tracking have also been compared with endoscopic and open surgical approaches for other orthopedic procedures, such as total hip and knee replacement, but no significant improvements have been reported to date in the reduction of associated complications.^23^^,^^24^

Various unmet clinical needs in orthopedic surgery could potentially be addressed by optical spectroscopy or imaging, which come in many different forms depending on the light–tissue interaction being sensed. They include diffuse reflectance that depends on the light absorption and (elastic) scattering of the tissue, optical coherence tomography (OCT) that images tissue microstructures and fluorescence and Raman (inelastic scattering) that report molecular signatures. These techniques may be implemented at the working tip of surgical instruments to help guide the procedure and minimize risk of complications. Both morphological and physiological information can be obtained in real time and in a noninvasive manner.

The full potential of photonic sensing in orthopedic surgery has not been explored but over the past few years there have been some developments for specific procedures and to refine surgical workflow. Examples include: a cranial perforator based on diffuse reflectance spectroscopy (DFS) that stops automatically at the dura,^25^ an intramedullary nail system with a DFS sensor^26^ that prevents overdrilling, and a pedicle screw insertion device with DFS guidance^27^^–^^30^ that facilitates spinal fixation. Although the first of these devices is used by neurosurgeons, the technology is relevant to orthopedic surgery, since similarly the objective is to avoid perforation through bone into critical adjacent normal tissues structures. While these examples illustrate the unmet clinical needs and the potential of optical techniques to provide greater control and reduced complications, work to date has been largely limited to preclinical or cadaveric studies.

There have also been only a few developments in the use of optical sensing for general orthopedic and spinal procedures, such as lumbar decompression, total hip or knee replacement, and shoulder replacement/fixation. These procedures utilize different surgical techniques/approaches into which optical guidance could be integrated, as summarized in Table 1. There are only limited data on the relevant optical properties of different layers of bone (periosteum, cortical bone, and endosteum). A recent study focused on percutaneous measurements of the optical absorption and reduced scattering coefficients of whole bone in the radius and distal tibia using a photon time-of-flight technique.^31^ This provided only average values of tissue, not specific to tissue layers, which may not be sufficiently reliable or accurate for optical sensing in critical locations (e.g., pedicle screw placement into cortical bone). This and other studies have shown strongly wavelength-dependent absorption and reduced scattering coefficients of bone in the range 0.1 to $0.5\;{\mathrm{cm}}^{-1}$ and 4 to $12\;{\mathrm{cm}}^{-1}$, respectively. Sekar et al. demonstrated a maximum CW penetration depth of between ∼7.5 and ∼15  mm at 785 nm with the lowest values measured at the trochanter due to strong scattering.^31^ In addition, the authors noted that at the penetration depths measured with their system in CW mode, they were objectively able to measure cortical bone at every position transcutaneously.^31^

**Table 1:** Overview of different surgical instrument methods potentially utilizing optical guidance.

Procedure	Surgical instrument methods
Total hip arthroplasty	Drilling, shaving, shaping
Total knee arthroplasty	Drilling, shaving, shaping
Shoulder fixation	Drilling
Lumbar decompression	Shaving, shaping
Pedicle screw placement	Drilling, screw insertion

With this brief background, the objective here is to explore the potential roles of optical sensing in specific orthopedic and spinal surgery applications, particularly to reduce complications by increasing surgical precision and to enable extension of the surgical field with greater control and improved safety. Moreover, we aim to provide insights to the optical sciences/engineering community as a stimulus for technology innovation, with particular attention to the inherent challenges in integrating optical sensing within delicate surgical instruments and to clinical applications that would benefit from the improved precision.

## 2 Optics-Enabled Orthopedic Technologies

### 2.1 Orthopedic Applications and Challenges

#### 2.1.1 Total hip arthroplasty

The first use-case of a drill incorporating optical sensing is for total hip arthroplasty, for which specific drilling locations are well prescribed to avoid damage to the neurovasculature.^32^^–^^34^ In general, the posterior-superior and posterior-inferior pelvic zones are considered “safe,” depending on the screw size. In general, screws <20  mm long should be used in the posterior–inferior direction to avoid the sciatic nerve, inferior gluteal nerve and blood vessels, as well as the internal pudendal nerve and vessels. However, there are some cases where either the iliac fossa has been damaged or the prescribed safe zones are too weak to anchor the acetabular implant. In these cases, the surgeon needs to drill in a direction where the pelvic bones are much thinner than the iliac fossa, so that there are vessels and nerves that could be damaged if the bone is pierced. However, these alternative approaches of drilling into the anterior–superior and anterior–inferior direction risk damaging the external iliac artery and vein (in the anterior–superior direction) or the obturator nerve, artery, and vein (in the anterior–inferior direction).^34^

**Fig. 1:**
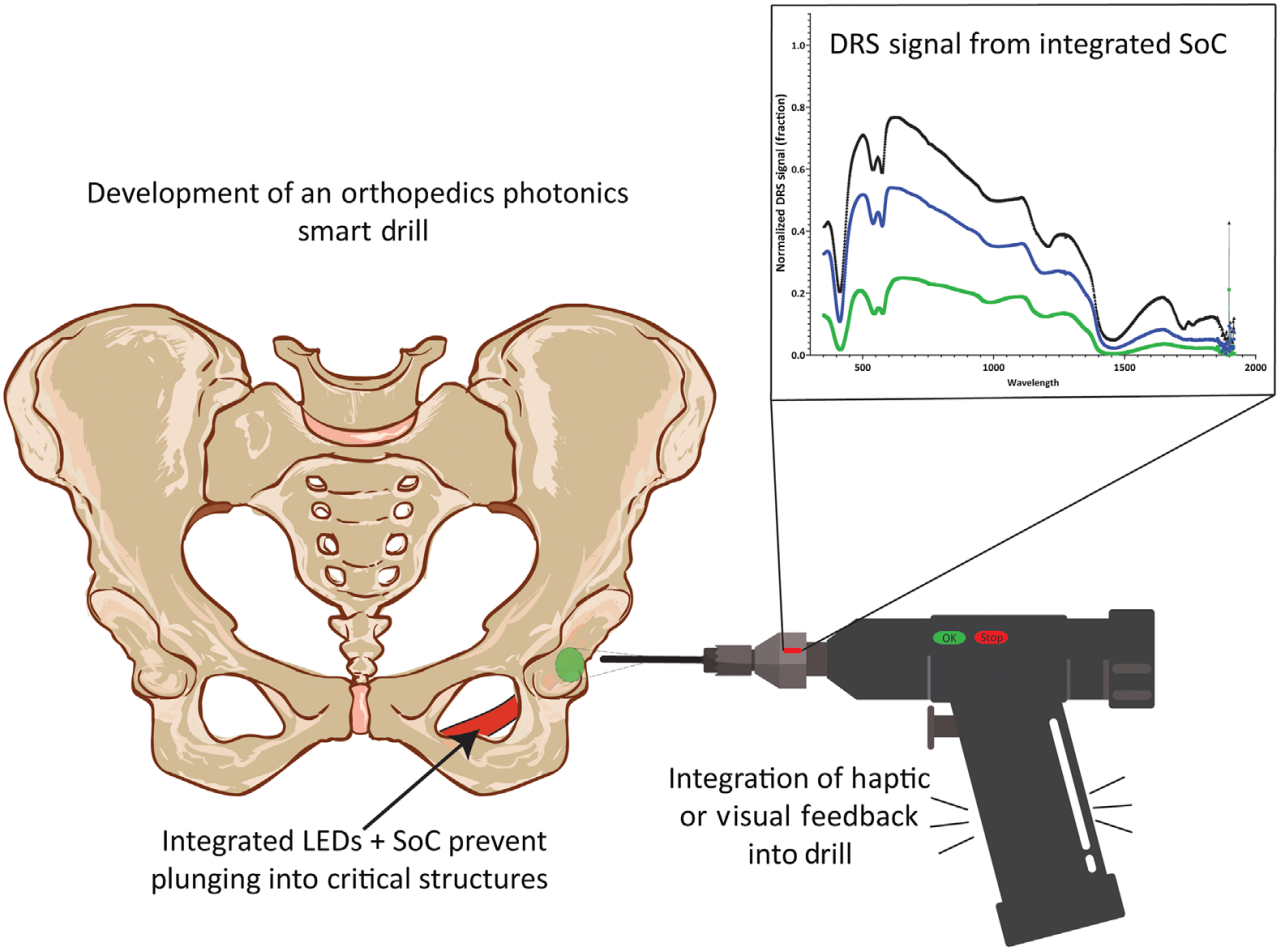
Concept of a “smart” optical drill for total hip arthroplasty.

Currently, the bone is drilled using haptic and/or auditory feedback to determine if bone/tissue boundary is being approached. In addition, clinical experience together with preoperative imaging inform the best path, and the drill and screw lengths should be chosen for whichever acetabulum quadrant was selected for optimal fixation. Other competing techniques such as navigation, intraoperative imaging, and robotics have been demonstrated, although their added benefit has not been established in total hip arthroplasty as compared with spinal approaches (laminectomy, pedicle screw placement) and knee arthroplasty: further randomized control studies are ongoing.^5^^,^^20^^,^^23^^,^^24^ In this complementary hybrid approach, development of an optics-enabled drill would act as an added safety device to warn of upcoming danger through feedback to the surgeon. This would not necessarily require automatic shut-off capabilities such as have been implemented in some orthopedic and cranial drills.^25^ We suggest that an optical sensor within the drill tip would report on whether the drill is encountering blood vessels and/or nerves and also sense the bone/tissue interface to warn of potential periosteum breach. This would increase confidence in utilizing the higher-risk quadrants without increasing complications. An example of how this drill could work is shown in Fig. 1.

There are, however, several challenges to this approach. Despite previous demonstrations of photonic devices integrated into solid drill bits such as cranial perforators,^21^ the bits used routinely in hip arthroplasty are flexible. They have a hollow core, which is well suited for fiberoptic placement, but large-core multimode glass fibers that are commonly used for such sensing have limited bend radius. More flexible plastic fibers might be an option but may have limited lifetime in the harsh drilling environment.

#### 2.1.2 Clavicle fracture fixation

In clavicle fracture fixation, rare (<1% of cases) but serious and life-threatening complications due to drilling into the subclavian neurovascular bundle have been reported.^35^ As in total hip arthroplasty, the procedure follows prescribed safe drilling angles and zones,^36^ despite which over-drilling still occurs, leading to possible laceration of the subclavian artery, damage to the brachial plexus or damage to the subclavian vein (not always due to the over-drilling itself but subsequent piercing by screws). This last complication may not be obvious during the procedure itself but leads to significant morbidity and need for revision surgery. Symptoms may not present for upwards of a decade, at which time claudication and critical limb ischemia may occur.^35^ In addition, other complications such as pseudoaneurysm, subclavian arteriovenous fistulas, air embolism, and severe postoperative radicular pain can occur that may not present for months or years.^35^

Most of the cases reporting damage to the associated vessels and nerves are in medial clavicle fixations, so that optical sensing could be used in these critical cases on the medial aspect of the clavicle where injury can occur. The most common cause of injury is overdrilling or taking an oblique approach through the clavicle instead of the prescribed superior–inferior direction. This can be exacerbated by portions of the subclavian vein adhering to the inferior border of the clavicle. It would then be of value if optical sensing could provide both feedback and a failsafe mechanism upon detecting imminent inferior breaching to stop the drill. This approach has been demonstrated with other optically enabled surgical devices.^25^^,^^26^ In addition to challenges similar to those in total hip arthroplasty, the screws and drill depth for medial clavicle fixation are relatively short, typically <18  mm in adults.^31^ One technical challenge, which is found across all the applications, is selecting the optimum optical properties for differentiating bone from surrounding soft tissues, especially where there may be vessel or nerve adhesions to the bone. Distinguishing between mixed fascia/muscle, blood, and nerves may be needed.

#### 2.1.3 Pedicle screw placement

Spinal surgery presents several scenarios where optical sensing could be incorporated, one being pedicle screw placement, in which drilling (as is commonly performed with percutaneous approaches) is technically challenging and complications from incorrect drilling have significant morbidity.^13^^,^^37^^,^^38^ The challenge in this application is in maintaining the correct trajectory within pedicular cancellous bone and avoiding excessive medial/lateral trajectory or, less commonly, anterior/superior/inferior trajectory that may result in cortical breaching. Deviating from the prescribed surgical path will inherently carry risk into injuring critical structures such as nerves or causing an incidental durotomy with a resultant cerebrospinal fluid (CSF) leak.^38^ Deviation from the planned trajectory may not be detected until after the screws have been inserted. A number of imaging modalities, including intraoperative fluoroscopy, CT and infrared navigation with tool tracking, have been reported for this application, with recent studies also involving robotics.^2^^,^^4^^,^^15^^,^^19^ The aim is to aid the surgeon in correct placement as well as getting the maximum purchase from the pedicle screws to minimize the risk of revision surgery, which is complex, invasive, and has higher complication rates and poorer outcomes, especially where screws have to be removed and repositioned. Optical sensing could play a complementary role in these procedures. A number of methods and approaches have been developed using optical sensing for percutaneous pedicle screw fixation, including integrating optical fibers within Kirschner K-wires, (a sharpened steel pin utilized for skeletal traction of the pedicle screw), to measure the diffuse reflectance, and photoacoustic imaging probes.^23^^–^^26^ Optical sensing could be used locally in conjunction with systems such as navigation and global CT guidance to provide a second check so the prescribed drill path is maintained in the cancellous bone. One option is to integrate sensing onto the navigation drill guide rather than into the drill bit itself and then coregister the navigational and optical signals. A second option would be to have the sensor at the tip of the drill to distinguish cancellous from cortical bone, alerting the surgeon to deviation from the cancellous bone trajectory.

Unlike some of the other orthopedic applications, a common overdrilling problem in the spine relates not to the forward-facing boundary but rather to medial and lateral breaches. This presents a three-dimensional navigational challenge to maintain the appropriate trajectory. The reasons for this difference compared to total hip arthroplasty or clavicle fixation are due to the nature of screw placement. In the case of pedicle screw placement, the screw has a fixed prescribed length with the goal of insertion into the vertebral body. For an anterior breach, the screw would have to go through the entire vertebral body, where 2-mm deviations can lead to medial/lateral breach (the most common breach orientations), or there is a possibility of an in/out lateral breach where the screw leaves the pedicle and the tip reinserts into the vertebral body. Superior/inferior breaches also occur albeit at a lower frequency with inferior breaches considered as serious as medial breaches given the close course of nerve roots along the medial and inferior pedicle borders.^39^

#### 2.1.4 Lumbar decompression

Degenerative spinal stenosis is a common condition, with reported symptomatic cases of ∼9% in adults within a Japanese population and increasing with age.^40^ The typical intervention is removal of the vertebral ligaments and laminae to relieve pressure on the spinal canal and nerve roots as they exit the spine. In surgically managed cases, there are different approaches for decompression, based on clinical judgment and the degree of stenosis, but can include either laminectomy or laminotomy. These procedures can be performed through open or minimally invasive techniques. Especially for the latter, there is a significant surgical learning curve and there are complications even for the fully trained surgeon.^10^^,^^41^^–^^43^ One of the most common complications is dural laceration, i.e., incidental cutting of the dura by the burr or other device (e.g., Kerrison or Cloward rongeurs—used to cut and pull away pieces of the lamina). This may or may not cause CSF leakage, with laceration rates reported in 15% to 20% of cases.^44^^,^^45^

**Fig. 2:**
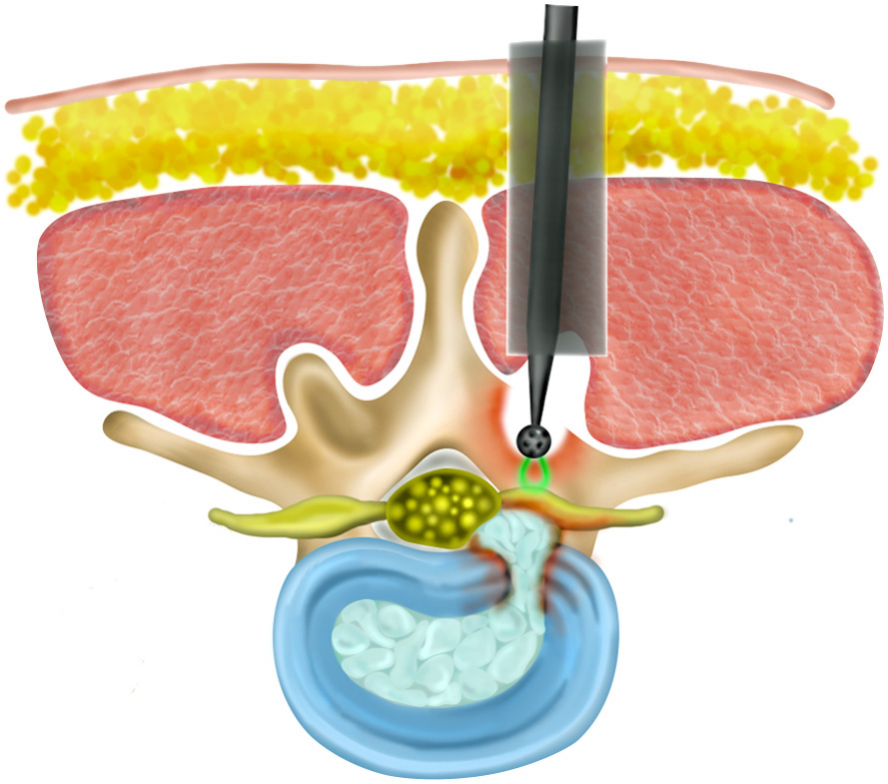
Concept of an optics-enabled burr for laminectomy.

In the minimally invasive approach, the operative field is viewed by endoscopy or microscopy, while open procedures provide direct vision. Robotic approaches have not been widely adopted to date.^46^^,^^47^ We envision optical guidance to prevent dural laceration, either by informing the surgeon when the dura is being approached or by stopping the device if imminent breach is probable by differentiating between bone and dura/CSF. This would also aid less experienced surgeons, shortening procedure times, and increasing confidence. An example of how this might work is presented in Fig. 2.

There are multiple technical challenges in this clinical application. First, the procedure itself is distinct from the other drilling procedures as the burr is used to shave back and forth to remove bone. Hence, the drill tip is not fully in contact with different pieces of bone, causing optical signal variations due to bone heterogeneity. Second, the geometry and size of the burr, which is spherical with diameter as small as 4 to 6 mm, make it more difficult to integrate fiberoptics. In the case of the hip, clavicle, and pedicle screws, the devices are large enough to carry the required number of fibers, but this is not the case for this procedure. It may then be necessary to add an accessory probe to provide illumination or detection separate from the drill bit itself, for example, using a wide-field imaging modality interfaced with the existing endoscopic system.

### 2.2 Integration of Optical Devices into Orthopedic Tools

**Fig. 3:**
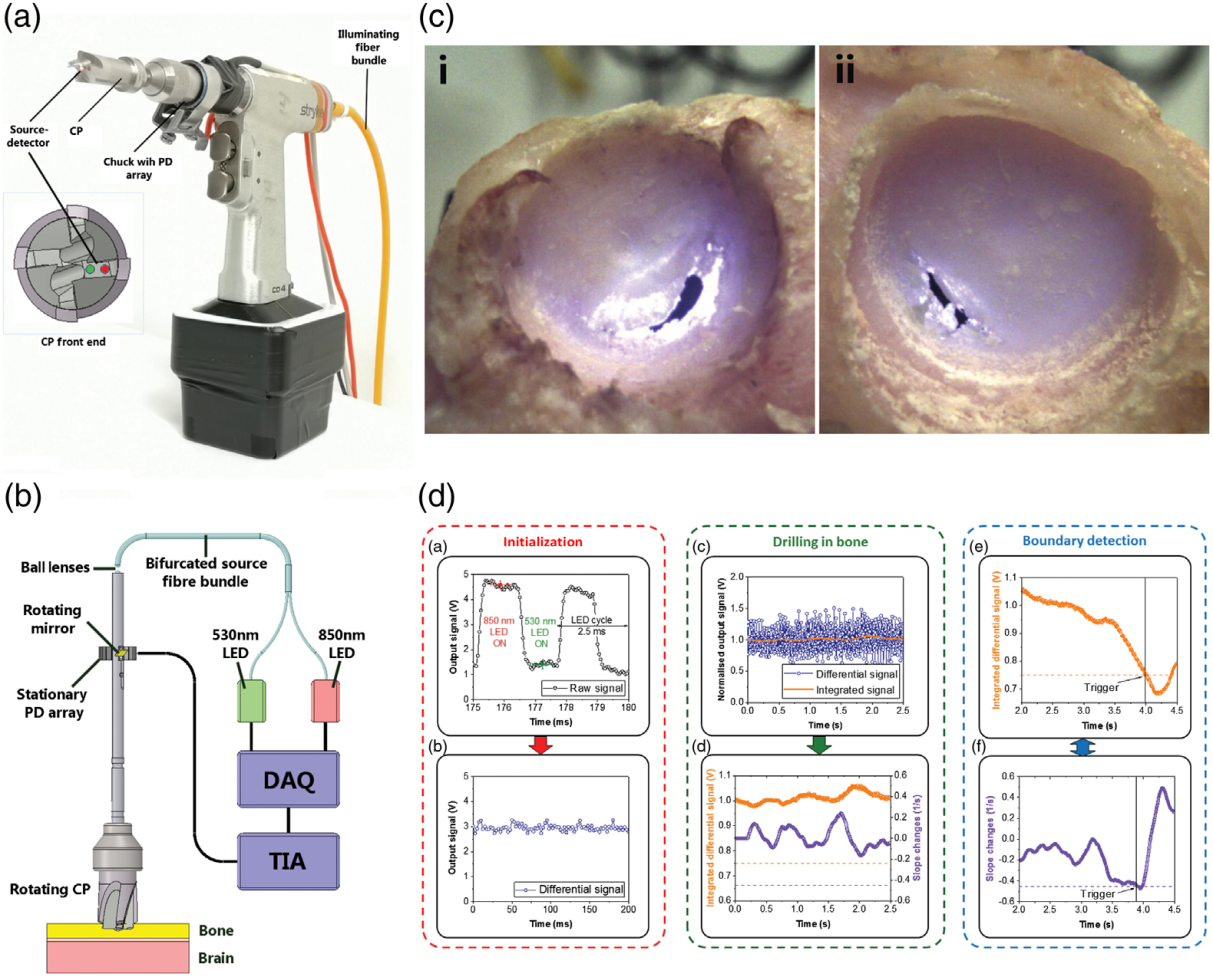
Implementation of two-wavelength DRS in a cranial perforator surgical drill bit to prevent plunging events during craniotomy. (a) Prototype of Stryker CD 4 drill with integrated DRS, (b) schematic of the integrated illumination and detection systems, (c) demonstration of successful stop of the drill in *ex vivo* (sheep) cranium using optical detection only, leaving ∼0.5-mm thick bone shelf just before the dura, (d) flow chart of 530- and 850-nm reflectance data acquisition and processing to detect the brain-bone boundary, applying threshold and slope algorithms. (Adapted from Ref. 25.)

In the above applications, an optical device and algorithms would use native optical properties of bone and surrounding tissues to aid the surgeon in avoiding overdrilling through bone or into critical structures, either by providing feedback or by automatically stopping the drill. Similar approaches have been reported in neurosurgery where, for example, a cranial perforator system was developed to sense the approaching dura and stop the drill prior to piercing the skull.^25^ Optical fiber-integrated pedicle screws have also been reported utilizing DFS.^27^ Integration of optics into a K-wire highlights a similar approach.^29^ The integration of optics within an orthopedic surgical drill that has been utilized preclinically is shown in Fig. 3. These first advances have shown the promise of optical sensing as a low-cost, noninvasive method to improve the accuracy and safety of orthopedic procedures. Table 2 highlights some examples reporting use of such technologies.

**Table 2:** Reported approaches of optical integration into orthopedic procedures.

Procedure	Clinical challenge	Typical guidance	Optical technique	Verification method	Detected structure	Ref.
Cranial perforation	Penetrating into brain	Mechanically clutched burr	Diffuse reflectance	*Ex vivo* animal	Bone/brain interface	25
Intramedullary nailing	Breach and soft tissue damage	X-ray	Diffuse reflectance	*Ex vivo* animal	Bone/muscle interface	26
Pedicle screw placement	Lateral and medial breach	CT	Diffuse reflectance, photo-acoustics	*Ex vivo* human	Cortical/cancellous bone	27–30

The use of native optical properties of tissues necessitates either a standard set of optical properties across a wide spectral range for the tissues of interest or a method of determining patient-specific tissue properties prior to drilling. Databases of optical properties have been generated for various tissue types to use as input to Monte Carlo simulations for photodynamic therapy (PDT).^48^ Similar to an approach used during PDT for prostate cancer, in which light penetration is measured to continually update optical absorption and scattering properties for dynamic dosimetry,^49^ real-time measurements could be employed to guide the surgeon as the drill moves into different tissue types. It may also be possible to employ a similar approach based on preoperative imaging, where patient-specific bone thickness could be used with varying muscle/fascia, blood, nerves/spinal cord, or CSF “background” tissues as a training set. However, this is nontrivial because of the large inter- and intrapatient variations in optical properties, as reported, for example, for bone^31^^,^^50^^–^^55^ and CSF.^55^^–^^57^ Hence, it may be difficult to achieve the accuracy needed without an intraoperative method of measuring the optical properties in individual patients. An alternate approach would be to collect a large data set to train an artificial intelligence (AI) algorithm that would subsequently be used for guidance.

Although this discussion has focused on the use of DFS and corresponding optical properties for detection of tissue boundaries or critical structures, other optical modalities could be integrated as alternative or complementary modalities. Raman spectroscopy, for example, has been used preclinically to detect osteoarthritic changes in human cartilage^58^ and transcutaneous *in vivo* detection of disordered bone.^59^ Raman, or possibly coherent anti-Stokes Raman (CARS) spectroscopy, could identify intrinsic biochemical “signatures” such as collagen between layers of bone, elastin/blood in vessels or myelin/lipids within nerves. Tissue autofluorescence or the use of exogenous fluorophores could be added for tissue discrimination, as increasingly used in guiding tumor resection. ^60^^–^^62^ This would avoid the added technical complexity and costs associated with nonlinear techniques such as CARS or, to detect collagen, second harmonic generation. (A caveat in using collagen detection for guidance is that it is present in multiple tissue layers and at boundaries between bone/periosteum or bone/cartilage/dura in vertebrae.)

In general, regardless of the optical technique and depending on the procedure, detection of the following structures will be needed: bone (cortical, trabecular, osteoporotic), soft tissues (muscle, subcutaneous tissue, dura), connective tissues (ligaments, cartilage), and neurovascular tissues (spinal cord, nerves, blood vessels, CSF). Even with some canonical training sets of optical properties, additional perioperative or intraoperatively measurements will likely be needed, using techniques such as those listed in Table 3. However, some of these are either time-consuming or only acquire superficial optical properties which limits their utility.

**Table 3:** Reported methods to measure *in vivo* optical absorption and elastic scattering properties of human tissues.

Method	Tissue type	Wavelength (nm)	Ref.
Spatially resolved reflectance measurements	Esophageal (normal, benign, malignant)	630	63
	Brain and bladder	420–450, 532, 635	64
	Esophageal wall	630	65
	Skin and underlying tissues	400–1050	66
	Common nevi, dysplastic nevi, and malignant melanoma skin lesions	483–917	67
	Prostate	732	68
Time-resolved reflectance measurements	Skin, subcutaneous fat and muscle	830	69
	Bone	760, 600–1200	31, 70
Spatial frequency domain imaging	Skin	450–800, 950–1600	71
	Ovarian tissue	730	72
Low-coherence enhanced backscattering spectroscopy	Duodenal mucosa	Tunable xenon lamp	73

In addition, any optically enabled tool would need an accuracy at least equivalent to existing clinical devices, typically sub-mm for most of the above applications. For total hip arthroplasty and clavicle fracture fixation, the required accuracy would be ≤0.5  mm. Although 0.5 mm would exceed many of the leading navigation systems and beyond what current systems may manage we propose this value as a benchmark for these specific indications where navigation is not routinely used and where surgeons want to be able detect specific nerves or blood vessels. A larger uncertainty would be clinically acceptable for applications in the spine, specifically for pedicle screw placement (as reflected in Table 4), although for lumbar decompression there is currently no competing technology that reduces dura laceration rate. State-of-the-art navigation systems for pedicle screw placement have reported deviations of <1  mm in screw location compared to postoperative CT imaging in the lateral and axial directions:^16^^,^^74^ even if the accuracy of optical sensing only matched that of navigation and intraoperative CT, this should be beneficial in a number of ways. First, reduction in radiation dose within the OR would benefit both patients and operators. Second, optical sensing could allow for real-time imaging and feedback, which current CT navigation systems cannot achieve, and should entail lower equipment costs that enable wider dissemination. However, recent studies involving optical sensing in neurosurgery, such as the cranial perforator, have suggested that sub-mm optical accuracy is attainable as the group could reliably stop the cranial perforator within 0.5 mm from the surface of the dura routinely, albeit in an *ex vivo* setting.^25^

**Table 4:** Drilling parameters and competing technologies.

Procedure	Drill type	Speed (rpm)	Feed/shaving rate (mm/s)	Accuracy required (mm)	Estimated forward sensing required (mm)		Competing approaches
					Auto stop	Surgeon cues	
Hip arthroplasty	Orthopedic drill with flexible drill bit	Up to 75k	4 to 5	0.5	1	2	Safe zones^a^, navigation, robotics
Shoulder fixation	Orthopedic drill with flexible drill bit	Up to 75k	4 to 5	0.2 to 0.5	0.5 to 1	1.5 to 2	Drill guides, safe zones
Pedicle screw	High speed drill with drill guide	Up to 90k	4 to 5	0.5 to 1.0	1 to 1.5	2 to 3	Navigation, CT guidance, fluoroscopic guidance
Laminectomy	High speed drill with flex-burr in some cases	Up to 90k	1 to 2	0.1 to 0.3	0.25 to 0.75	1 to 1.5	Endoscopic + microscopic imaging, robotic (open decompression^b^)

a Safe zones are prescribed areas of tissue where procedures can be performed with minimal risk due to the absence of neurovascular structures.

b Open decompression refers to procedure via a full skin incision where pressure on the spinal cord is relieved by removing the entire posterior portion of the vertebrae (the lamina).

With these various factors in mind, we suggest the following requirements for optical sensing in orthopedic surgical devices.

• Field of view: 1.5 to 2× the drill tip/burr diameter;
• Depth of view: either 2× or 3× the depth accuracy required (for autostop or surgical cues, respectively);
• Spatial resolution: 0.1 mm by 0.1 mm (to differentiate neurovasculature from surrounding tissue);
• Sensitivity: ∼90% to 95% (comparable to that of CT for pedicle screw placement);
• Specificity: ∼70% to 80% (variable, depending on procedure and surgical experience).

A further consideration is the optical signal sampling rate required in each procedure, as summarized in Table 4, taking into account also the signal integration and analysis time and the time to stop the drill. We have provided estimates of the forward-sensing distance required, depending on whether autostop is implemented or the device is passive and simply alerts the surgeon. For the hip and shoulder procedures, sub-mm accuracy is required but the total drill time is only ∼5  s so the high rotational speed and axial feed rate may generate various forms of noise (vibrational, thermal from friction, and mechanical from the fiberoptic coupling) that may degrade the accuracy. A further consideration is that some procedures would require constant contact between the optical fiber and the tissue to maximize the signal.

The final aspect of integrating optical sensing into orthopedic surgical tools is the interaction with the surgeon. In general, providing visual and/or audio cues is likely safer and would be more easily adopted than autostop, at least initially. For example, visual cues could comprise green/red LEDs to indicate when the bit tip is still in bone or approaching a boundary, respectively. Alternatively, a single LED could be set to blink only when a boundary is being approached. Audio cues such as beeping are interesting in that many surgeons already use audio feedback to determine when they have moved into different types of bone, based on the changing pitch of the sound made by the drill bit moving toward a boundary. Haptic feedback is another option that has been trialed in surgical robotics,^75^^–^^77^ although vibration of the tool is a challenge.

These various approaches could be integrated with other-modality devices and/or within robotics devices for MIS. An example is integrating optics within a pedicle screw that already utilizes neuronavigation or CT imaging. Communication between the modalities could be done wirelessly or using data cables coming off the drill (see Fig. 3). Semiautomated OCT imaging has been already tested preclinically in an ophthalmic surgery robotic system.^78^ A long-term goal would be optics integration with AI-enabled robotics for autonomous or semiautonomous surgery, analogous to CyberKnife radiation systems^79^ or the RAVENII robot for brain tumor ablation.^80^

A final aspect of the integration of optics into surgical devices is the possibility of changing the surgical approach for many of these procedures through opening avenues that were previously deemed too risky. For example, if critical neurovascular structures could be reliably detected, it would markedly alter current procedures such as laminoplasty or enable a direct anterior approach in total hip arthroplasty. Likewise, new approaches would be enabled in cranial procedures, transsphenoidal surgery, and shaving/oncological procedures.

## 3 Conclusions

Orthopedic and spinal procedures are extremely common and their use increases with population ageing. Here, we have considered a number of such applications to illustrate how integrating optical sensing into the surgical tools could reduce risks and complication rates, allow surgeons to more confidently approach anatomical sites that are inherently less safe (e.g., as in hip arthroplasty), shorten operation times, and even enable novel surgical procedures. We envision that the integration of optics within orthopedic tools would use primarily the native optical properties of bone and surrounding soft tissues to provide near real-time feedback on upcoming structures (Fig. 1) or signaling, by visual and/or auditory means, when only a thin layer of bone remains (Fig. 2). This will complement other techniques such as navigation to ensure that the prescribed surgical plan is achieved with maximum safety and efficacy.

The need for such additional guidance will only expand with evolving minimally invasive and percutaneous procedures, so there are significant opportunities for further development of optical devices integrated into orthopedic surgical tools.
